# Comparative Proteomics Profile of Lipid-Cumulating Oleaginous Yeast: An iTRAQ-Coupled 2-D LC-MS/MS Analysis

**DOI:** 10.1371/journal.pone.0085532

**Published:** 2013-12-26

**Authors:** Jiahua Shi, Huixing Feng, Jaslyn Lee, Wei Ning Chen

**Affiliations:** 1 School of Chemical and Biomedical Engineering, College of Engineering, Nanyang Technological University, Singapore; University Paris South, France

## Abstract

Accumulation of intracellular lipid in oleaginous yeast cells has been studied for providing an alternative supply for energy, biofuel. Numerous studies have been conducted on increasing lipid content in oleaginous yeasts. However, few explore the mechanism of the high lipid accumulation ability of oleaginous yeast strains at the proteomics level. In this study, a time-course comparative proteomics analysis was introduced to compare the non-oleaginous yeast *Saccharomyces cerevisiae*, with two oleaginous yeast strains, *Cryptococcus albidus* and *Rhodosporidium toruloides* at different lipid accumulation stages. Two dimensional LC-MS/MS approach has been applied for protein profiling together with isobaric tag for relative and absolute quantitation (iTRAQ) labelling method. 132 proteins were identified when three yeast strains were all at early lipid accumulation stage; 122 and 116 proteins were found respectively within cells of three strains collected at middle and late lipid accumulation stages. Significantly up-regulation or down-regulation of proteins were experienced among comparison. Essential proteins correlated to lipid synthesis and regulation were detected. Our approach provides valuable indication and better understanding for lipid accumulation mechanism from proteomics level and would further contribute to genetic engineering of oleaginous yeasts.

## Introduction

Energy shortage has become an urgent problem all over the world. Meanwhile, the global warming caused by excessive CO_2_ production from fossil fuels aggravates the situation [1]. Microbial production of high-energy fuels represents one of the viable options for sustainable energy supply due to its considerable advantages, such as being renewable, biodegradable and nontoxic. In addition, biofuel has attracted numerous attention during the past decade [2]. Biofuel consists of alkyl esters that can be derived from triacylglycerols (TAGs) or free fatty acids (FFAs) by transesterification or esterification, respectively [3]. Hence research into the accumulation of microbial lipids, constituted mainly by triacylglycerols (TAGs) [4] and fatty acids, is an important issue and increasingly catching the attention of more and more researchers.

Oleaginous microorganisms, such as fungi, yeast, bacteria and microalgae, are capable of accumulating lipid with a yield of more than 20% in their cells [5,6]. Oleaginous yeast has considerable advantages compared to other kinds of oleaginous microorganisms, such as high growth rate, high lipid accumulation ability, easy culture method and ability to be grown in conventional bioreactor [7]. The majority of lipids produced by oleaginous yeast are triacylglycerols, which could be easily converted into fatty acids. The rest of the components of lipids consist of a small quantity of free fatty acid together with other neutral lipids, sterols and polar fractions [8]. Apart from high productivity of lipids, the variety of substrates that oleaginous yeasts can utilize is also an enormous advantage. Carbohydrates, hydrocarbons and waste materials could be effectively utilized by oleaginous yeast and converted into lipids [9-11]. Hence, all these properties make oleaginous yeast gain more popularity compared with other oleaginous microorganisms.

Numerous yeast species are reported to be oleaginous yeasts, such as *Rhodosporidium toruloides* [12], *Trichosporon fermentans* [13], *Cryptococcus albidus* [14], *Yarrowia lipolytica* [15] *a*nd *Lipomyces starkeyi* [16]. They are able to accumulate lipids ranging from 25% to 70% under appropriate culture conditions, including correct selection of carbon source and nitrogen source, relatively high C/N ratio, proper temperature of cultivation, pH value, dissolved oxygen concentration and inorganic salts concentration [7]. 

The industrial profit of derived biofuels has led to more research, exploring the mechanism of the prominent ability of lipid storage in oleaginous yeast. Metabolic investigations suggest that under nitrogen-limited condition, a series of reactions would be activated in oleaginous yeast strains, contributing to a large scale production of acetyl-CoA, which is the precursor for fatty-acid synthesis [17]. Several key enzymes are also found to be crucial, for example, ATP-citrate lyase (ATP-CL) is found to be uniquely existing in oleaginous yeast strains and has never been found in non-oleaginous ones [18]. It could catalyze conversion of citric acid to acetyl-CoA and thus continuously provides basic subunit for fatty acid synthesis.

Although major metabolite pathways and some key enzymes have been determined, lipid accumulation mechanism is rarely reported from a proteomics level, which would contribute to a more comprehensive understanding. In this study, we performed a comparative proteomics profile of three yeast strains and explored the possible mechanism of lipid accumulation by comparing the protein profile between two oleaginous yeast strains and non-oleaginous strain during the various growth and lipid accumulation phase. *R. toruloides* was widely studied as industrial strain for lipid extraction and usage due to its high lipid productivity, which was reported surprisingly 67.5% of their dry cell weight [19]. *C. albidus* was reported to produce up to 46.3% (w/w) lipid [14] and chosen as a medium lipid content strain. In order to further identify the differences between oleaginous yeast and non-oleaginous yeast, *S. cerevisiae* was also selected to be a ‘control’, as it is considered non-oleaginous yeast, due to its low lipid content, which consists of no more than 15% of its biomass [20].

Two dimensional (2-D) LC-MS/MS analysis has been more preferable in proteomics profiling analysis due to its higher efficiency and accuracy comparing to the traditional 2-D gel electrophoresis, especially when coupled with iTRAQ labelling method. Isobaric tag for relative and absolute quantitation (iTRAQ) labelling method is a MS-based approach allowing multiplexing of up to eight samples and the relative quantification of multiple peptides of each protein, which relies on the derivatization of primary amino groups in intact proteins [21,22]. After labelling with different isotopes, the derived peptides in each strain would experience an identical mass and retention time during LC-MS analysis. The precursor ions are analyzed by the following MS/MS analysis. The intensities of reporter ions would relatively indicate the concentration of peptides correspondingly [23].

This study successfully set up a platform of proteomic profiling in yeast strains and compared protein expression differences at different growth and lipid accumulation stage. Proteins revealed stable and remarkable change in comparison and many of them revealed potential importance in lipid accumulation in further analysis. To our knowledge, this study was the first one comparing between non-oleaginous with oleaginous yeast strains on proteomics level using iTRAQ-coupled 2-D LC-MS/MS method.

## Materials and Methods

### Strains, Culture Conditions and Chemicals

The wild type yeast strain *S. cerevisiae* BY4741 was obtained from European *Saccharomyces cerevisiae* Archive for Functional Analysis (Euroscarf) and grown at 30°C, 250 rpm in nutrient-rich medium (YEPD, 10 g/L yeast extract, 20 g/L peptone, 20 g/L dextrose). The oleaginous yeast strains *R. toruloides* 10788 and *C. albidus* 56297 were purchased from the American Type Culture Collection (ATCC). Two-stage process was used for cultivation. Cells were first cultured in YEPD medium for proliferation until stationary phase. After that, cells were centrifuged, washed twice with distilled water and transferred with 10% v/v inoculum into nitrogen-limited culture medium (containing per litre: glucose 35 g, yeast extract 0.5 g, (NH_4_)_2_SO_4_ 0.1 g, KH_2_PO_4_ 1.0 g, MgSO_4_·7H_2_O 1.5 g, pH 6.0) to trigger lipid accumulation phase. Cells were maintained at 28°C and agitated at 250 rpm in both stages. The carbon to nitrogen (C/N) ratio in YEPD and nitrogen-limited medium was 4.5 and 135, respectively. All of the three strains were cultivated in 250 mL flasks and maintained at 4°C on YEPD agar (10 g/L yeast extract, 20 g/L peptone, 20 g/L dextrose, 20 g/L agar) plates and transferred to fresh plates every month. 

### Growth and Lipid Content Measurements

Optical density at 600 nm (OD_600_) was measured for growth record using Nano Drop 2000 (Thermo Scientific). In order to determine cell dry weight, 20 mL of cell culture was took and washed twice with pre-chilled distilled water, then lyophilized to constant weight in freeze drier. Extracellular glucose content was determined by anthrone-sulfuric acid method [24]. R. Schneiter and G. Daum’s method was used to measure total cellular lipid [25]. 

### Protein Extraction, Digestion and Labelling with iTRAQ Reagents

For cell lysis and protein extraction, all steps were carried out on ice to avoid denaturation of proteins. 20 OD_600_ units of yeast cells were collected by centrifugation at 10,000 rpm, 4°C for 5 min. The cell pellets were washed twice by ice cold water and supernatant was discarded. Lysis buffer containing the following compositions gave the highest and most stable protein level when treating the same amount and batch of cells: 8 M Urea, 50 mM DTT, 50 mM Tris-Cl (pH7.6), 100 mM NaCl, 0.1% Triton X-100, 1 mM EDTA and 1 mM PMSF. The pH value of lysis buffer was adjusted to 7.4 using 1 M HCl. Three hundred microliter lysis buffer and approximate 200 µL glass beads were added to the pellet and disruption was conducted in the homogenizer by shaking ten periods of 30 s with 2 min cooling intervals on ice. Lysate was centrifuged at 10,000 rpm for 10 min at 4°C and supernatant was transferred to clean tubes and stored at -80°C. 

Prior to further treatment, lipid contaminants in protein extract were removed by organic solvents according as demonstrated [26]. Protein concentration was determined using Quick Start Bradford Protein Assay (Bio-Rad) according to Bradford method [27] followed by precipitation by 2-D Clean-Up Kit (GE Healthcare). Sample preparation and iTRAQ labelling was carried out using iTRAQ kit from AB Sciex. Briefly, protein samples were re-dissolved in 20 µL 0.5 M triethylammonium bicarbonate and 1 µL 2% SDS. Afterwards, 2 µL 50 mM tris-(2-carboxyethyl)-phosphine was added to reduce the disulfide bonds of the proteins at 60°C for 1 h. Alkylation was carried out by adding 1 µL 200 mM methyl methanethiosulfonate to reversibly block cysteine group at room temperature for 15 min. Digestion of each sample was then processed at 37°C for 16 h with a final concentration of 10 ng/µL sequencing grade modified trypsin solution (Promega). Samples were labelled with the iTRAQ tags (AB Sciex) as follows: *S. cerevisiae*, iTRAQ 114; *C. albidus*, iTRAQ 115; *R. toruloides*, iTRAQ 116. The labelled samples were then combined together accordingly and desalted by C_18_ Cation-Exchange Cartridge system (AB Sciex) together with C_18_ Sep-Pak Plus Short Column (55-105 µm) (Waters) to clean up mixture and remove salt and contaminants. The sample was then lyophilized and dissolved with 5% acetonitrile with 0.1% formic acid to proper concentration.

### On-line 2-D Nano-LC-MS/MS Analysis

Agilent 1200 series nanoflow liquid chromatography system (Agilent Technologies) was interfaced with 6500 Q-TOF mass spectrometer with HPLC-Chip Cube (Agilent Technologies) for two dimensional protein analysis. The HPLC-Chip was a combination of Zorbax 300SB C_18_ reversed-phase column (75 µm x 50 mm, 3.5 µm) packing with Zorbax 300SB C_18_ enrichment column (0.3 x 5 mm, 5 µm) [28].

**Figure 1:**
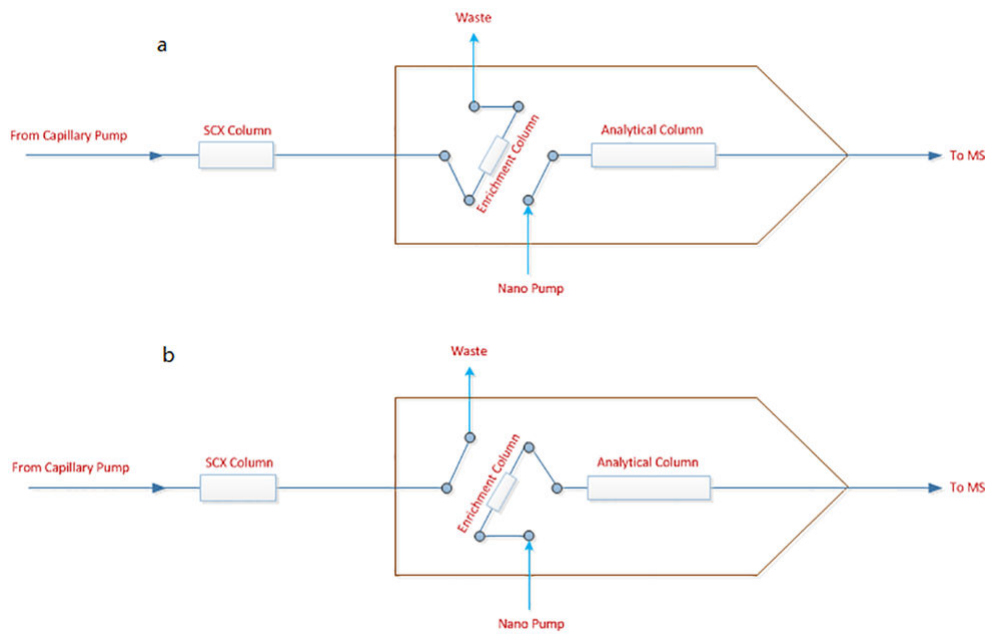
Workflow of on-line 2-D nano-LC. (a) The peptides eluted through SCX column and trapped onto enrichment column. (b) HPLC-chip was switched to analytical mode and the previously trapped peptides were eluted and analysed by analytical column.

In the first dimension, 4 µg combined peptide mixtures was loaded onto the PolySulfoethyl A strong cation-exchange (SCX) column (0.32 x 50 mm, 5 µm) and the retained peptides were stepwise eluted by sequential injection of 8 µL salt plugs with series of ammonium formate solutions in nine gradient concentrations of 20, 40, 60, 80, 100, 300, 500, 1000 mM, respectively. In the second dimension, the peptides were eluted through SCX column and trapped onto Zorbax 300SB C_18_ enrichment column during the enrichment mode by buffer A (5% acetonitrile and 0.1% formic acid) with a flow rate of 4 μL/min. Subsequently, the HPLC-Chip was switched to analytical mode and the peptides previously trapped on enrichment column were eluted for 60 min by buffer B (0.1% formic acid) and buffer C (a nanoflow gradient of 5- 80% acetonitrile + 0.1% formic acid) at a flow rate of 300 nL/min and further flowed through the analytical Zorbax 300SB C_18_ reversed-phase column for separation (Figure 1). The effluent was directly detected and analysed by 6500 Q-TOF mass spectrometer with a capillary voltage of 1950 V. In total, 10 runs were carried out to accomplish analysis of one sample. For MS analysis, positive ionization mode was used and survey scans were acquired from m/z 300 to 2000 with an acquisition rate of 4 spectra per second. Four most abundant ions that exceeded 1000 counts were selected for MS/MS analysis from m/z 50 to 2000. Control compounds with mass of 121.0509 and 922.0098 were used for automatically mass correction in each run.

### Data Analysis and Interpretation

Peptide quantitation and protein identification were performed using Spectrum Mill MS Proteomics Workbench (Agilent Technologies). Each MS/MS spectrum was searched based on species of microorganisms against the UniProt-Swiss-Prot database. Proteins qualified for further statistical analysis should meet the following criteria: two or more unique peptides identified with high confidence (>99%), protein score was more than 11.0, peptide score was at least 6.0 and the p value in Protein Quant was limited to be below 0.05. Methylmethanethiosulfate-labeled cysteine and iTRAQ modification of free amine in the amino terminus and lysine were set as fixed modification. Protein relative quantification using iTRAQ was performed on the MS/MS scans. It was represented by the ratio of peak areas of masses of the tags that correspond to the iTRAQ reagents, which was 114, 115 and 116 Da, respectively. By dividing the peak areas observed at 115.1 and 116.1 m/z by that at 114.1 m/z, the relative amount of a peptide in each sample was calculated. The ratios were corrected for overlapping isotopic contributions, estimating the relative abundances of a particular peptide. Three independent batches were performed to increase statistically evidence of protein expression. Proteins quantification with relative expression ≥1.2 or ≤0.8 was selected for further analysis.

## Results and Discussion

### Lipid Content Comparison

**Figure 2:**
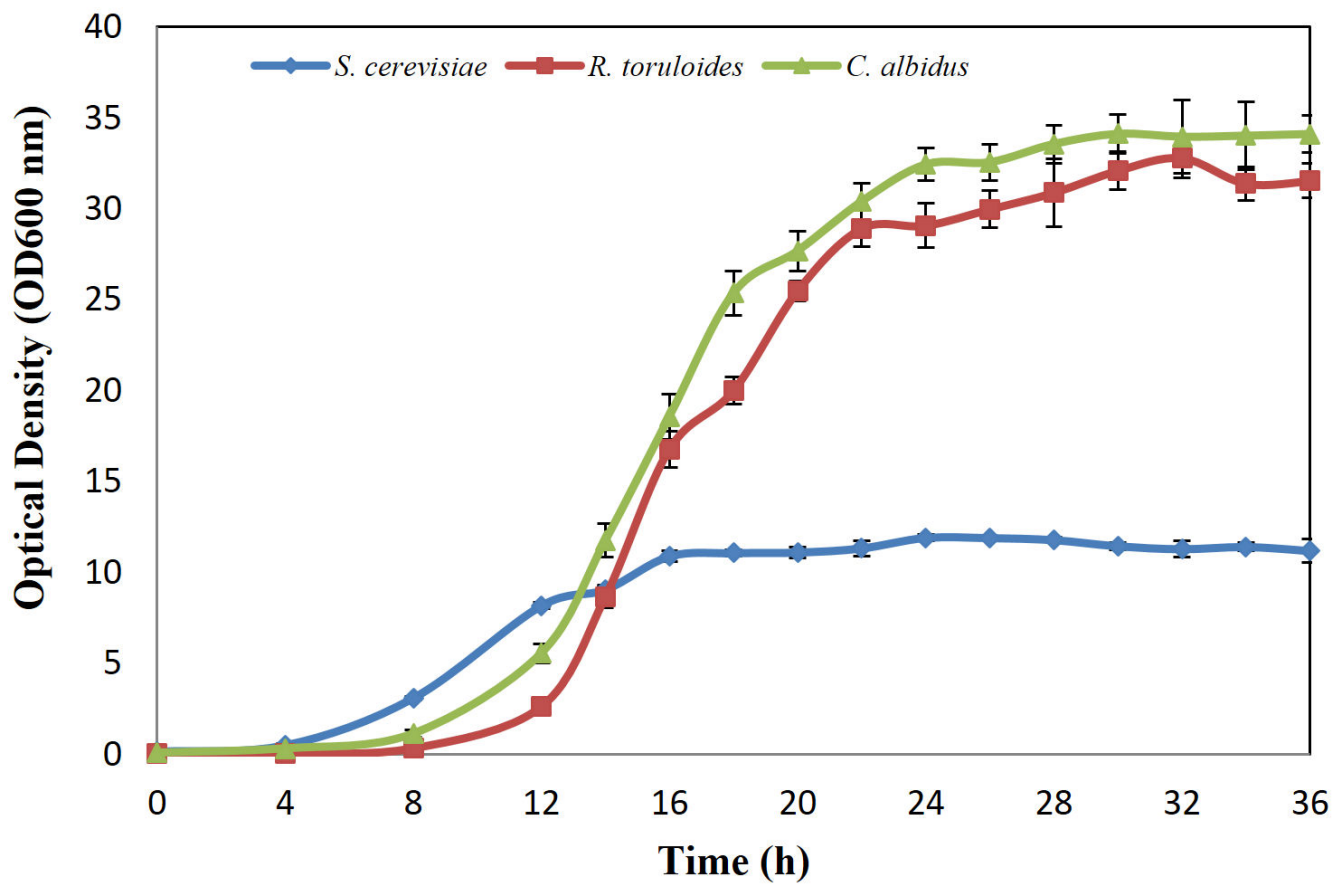
Growth curve of *S. cerevisiae*, *R. toruloides* and *C. albidus* in YEPD medium. Data from three replications were analyzed to calculate means and standard errors.

**Figure 3:**
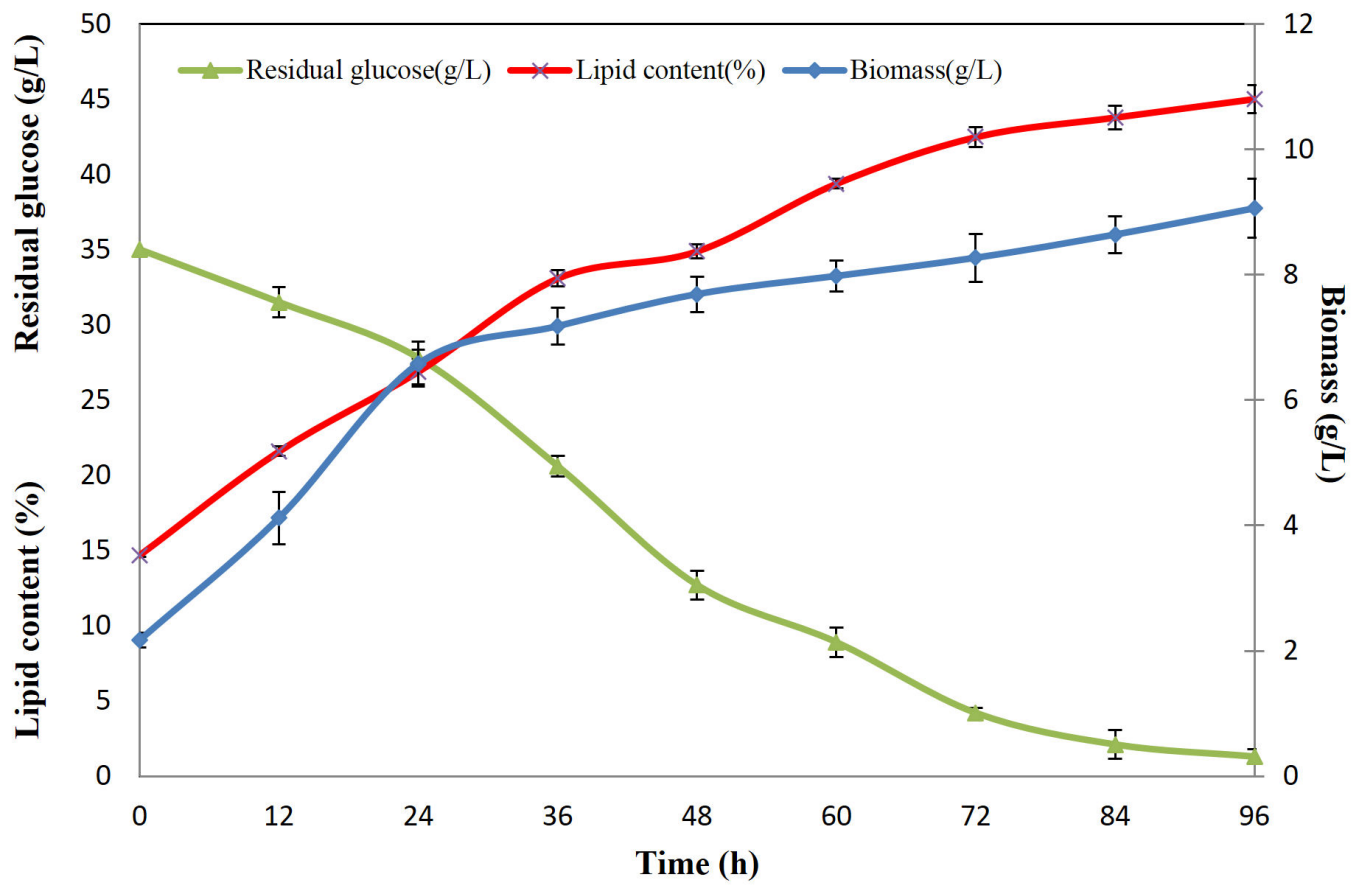
Substance level dynamic change of *R. toruloides*. Biomass (g/L), residual glucose (g/L) and lipid content (%) at different time points were presented.

**Figure 4:**
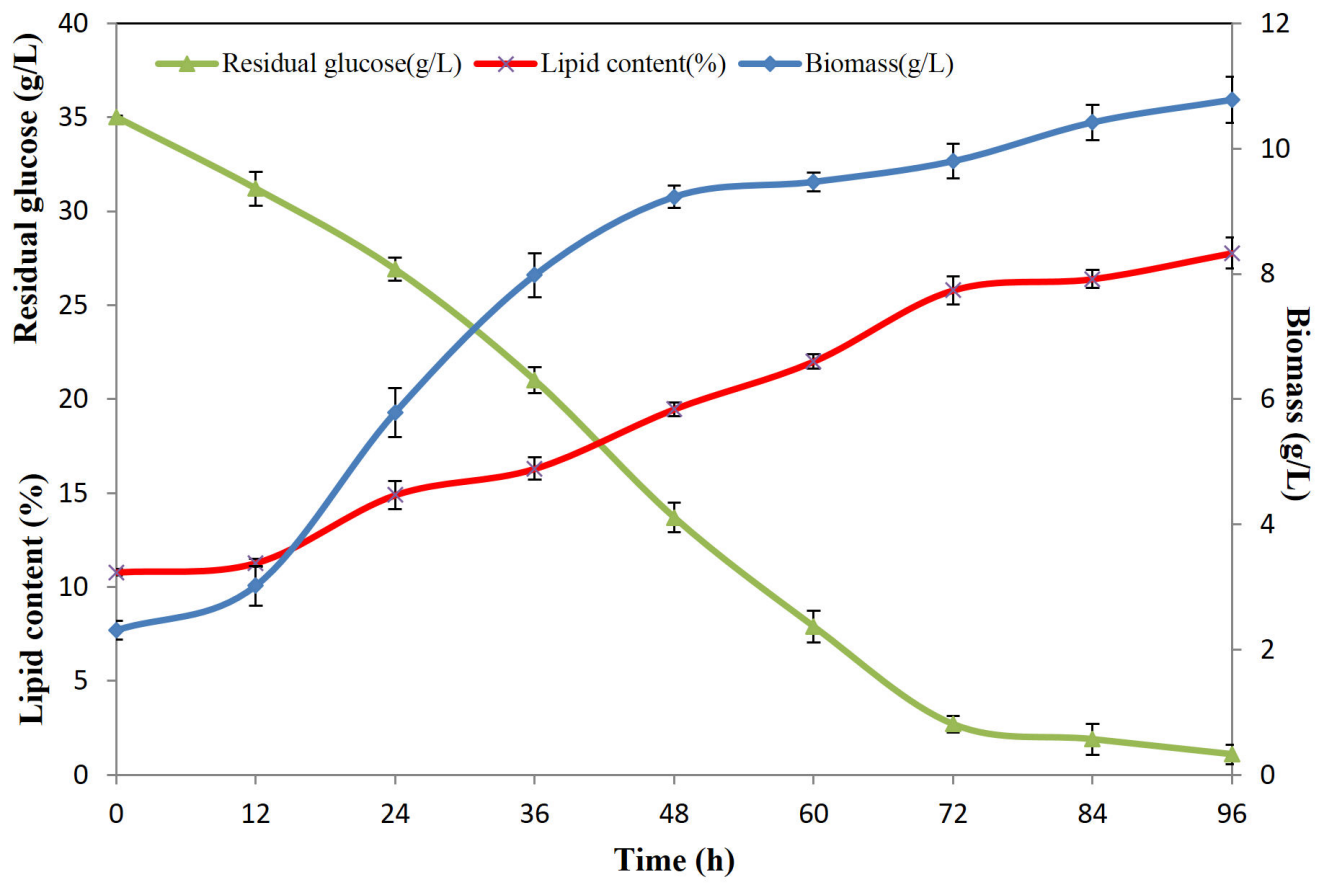
Substance level dynamic change of *C. albidus*. Biomass (g/L), residual glucose (g/L) and lipid content (%) at different time points were shown.

For *S. cerevisiae*, cells were cultured in YEPD medium and collected at early, middle and late lipid accumulation stages, which were 8 h, 18 h and 36 h, respectively (Figure 2). The lipid content was very low, reaching the highest level at 7.98% at 36 h. The two oleaginous yeast strains, *R. toruloides* and *C. albidus*, were cultured in YEPD medium (Figure 2) for proliferation, until the residual glucose approached zero at 30 h. The lipid contents were 14.7% and 10.73% respectively in this step. Then *R. toruloides* and *C. albidus* cells were centrifuged and transferred into nitrogen limited medium with a high C/N ratio of 135, to stimulate lipid accumulation. Samples were collected at 12 h, 24 h and 96 h, which represented early, middle and late lipid accumulation stages. *R. toruloides* reached a cell density of 9.08 g/L and lipid content of 45.03%, when glucose was exhausted at 96 h (Figure 3). *C. albidus* showed the ability to grow to a cell density of 10.78 g/L and accumulate lipid up to 27.74% of dry cell weight at 96 h (Figure 4). The data above clearly showed lipid accumulation differences between the non-oleaginous yeast *S. cerevisiae*, and oleaginous yeast strains *C. albidus* and *R. toruloides*. Although the lipid content of each strain was lower than that previously published in literature, it might be due to the fact that the cells were cultured using bioreactors and fed-batch method, as compared to shake flasks in a laboratory scale in this study, which is sufficient for proteomic study.

### ITRAQ Analysis and Protein Identification

Protein extraction was difficult due to the rigid yeast cell wall and it took effort to maintain the activities of proteins, as they were easily denatured when treated too harsh during cell wall disruption. Considering that the lipid content was high in oleaginous yeast strains, a higher concentration of denaturing reagents was used in the lysis buffer to help efficiently break lipid-lipid and lipid-protein interactions and increase protein solubility. Excess addition of detergent Triton X-100 in lysis buffer successfully overcame this problem without denaturing the proteins, as Triton X-100 was more efficient at breaking lipid-lipid and lipid-protein interactions, rather than protein-protein interactions [9,29]. Agitation with glass beads could efficiently break the cells with proper lysis buffer, but the excess lipid in oleaginous yeast cells affected the protein charge and molecular weight seriously as they would bind proteins through hydrophobic interactions, resulting in insoluble protein-lipid complex formation. Hence organic solvents treatment was further used to thoroughly remove the lipid contaminants. In the presence of chloroform/ methanol (2:1, v/v), the combination of 10%TCA/acetone and aqueous TCA wash steps showed effective removal of lipids. After extraction and lipid removal, protein samples were quantified and digested into peptides and labelled as follows: *S. cerevisiae* with iTRAQ 114; *C. albidus* with iTRAQ 115 and *R. toruloides* with iTRAQ 116. The three strains were compared in the following 3 phases: early lipid accumulation stage comparison (*S. cerevisiae* 8 h, *C. albidus* 12 h in nitrogen limited medium, *R. toruloides* 12 h in nitrogen limited medium); middle lipid accumulation stage comparison (*S. cerevisiae* 18 h, *C. albidus* 24 h in nitrogen limited medium, *R. toruloides* 24 h in nitrogen limited medium) and late lipid accumulation stage comparison (*S. cerevisiae* 36 h, *C. albidus* 96 h in nitrogen limited medium, *R. toruloides* 96 h in nitrogen limited medium). Three independent experiments of protein profile by 2-D nano-LC-MS/MS analysis were conducted for each group of sample. 

### Differentially Expressed Proteins within Comparison

Differential protein expression patterns were assessed through analysing the changes in protein expression among comparison of different lipid accumulation stages of *S. cerevisiae*, *C. albidus* and *R. toruloides*. The identified proteins were classified into eight functional groups: metabolism, biosynthesis, transportation, signal transduction, stress response, structural proteins, ribosomal proteins, and unclassified proteins with other functions. 

**Figure 5:**
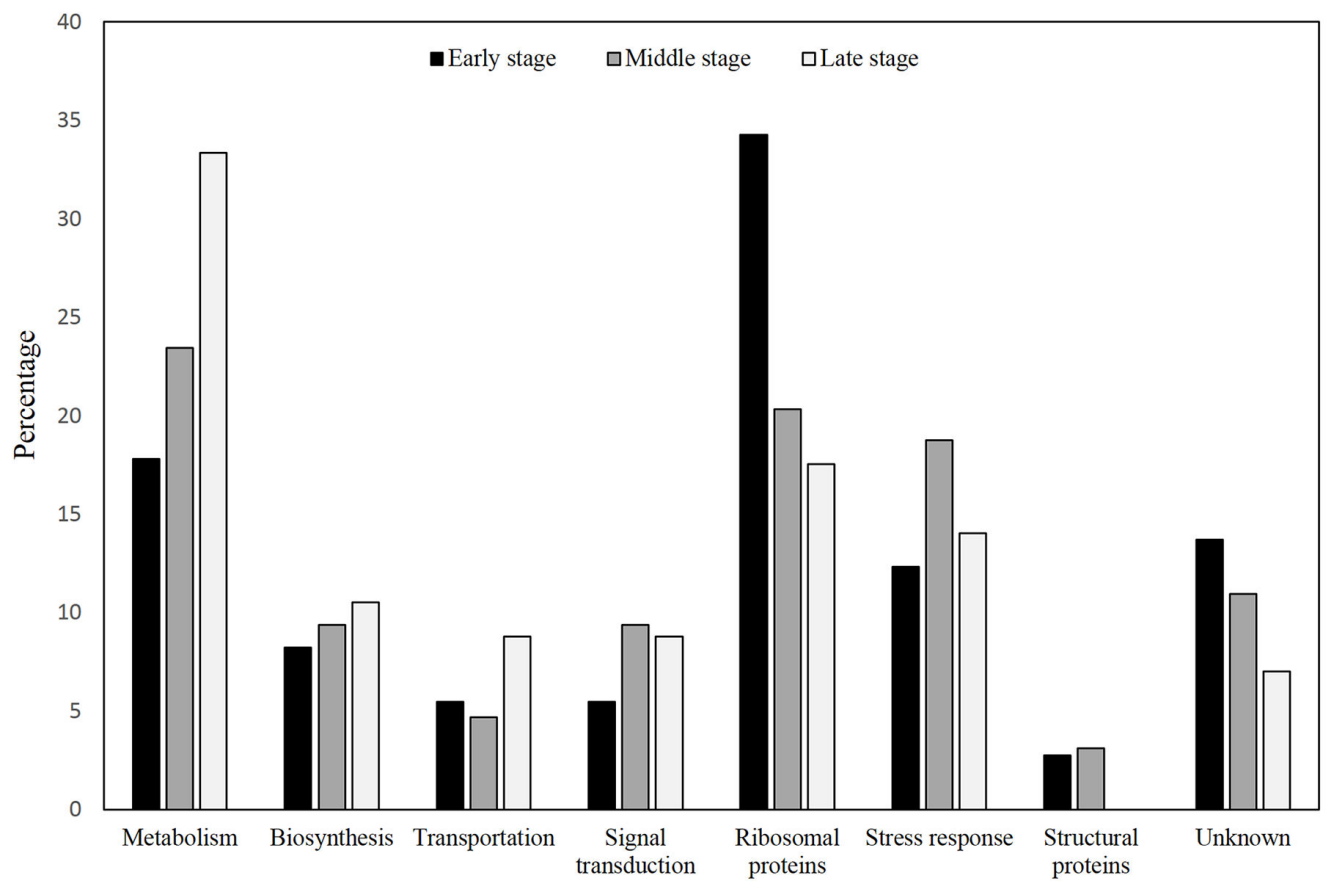
Functional distribution of proteins identified during each lipid accumulation stage. Proteins were classified into eight groups according to their different functions. The percentage of proteins of each function out of the whole proteins identified at each stage were exhibited.

**Figure 6:**
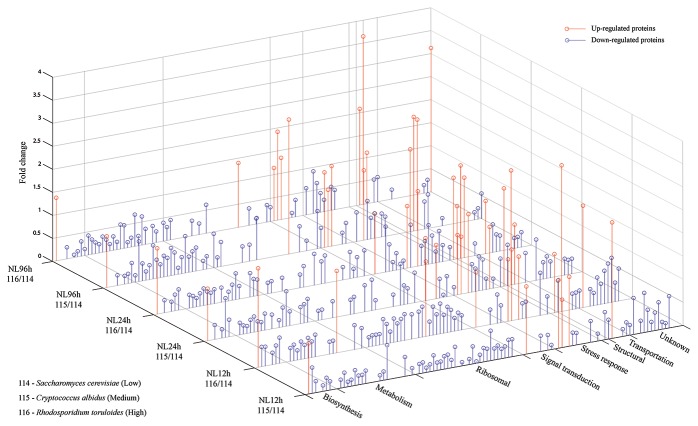
Fold change of proteins of different functional distribution at 12h, 24h and 96h. ‘NL’ represented nitrogen limited medium; red circles represented fold change of up-regulated proteins; and blue circles represented fold change of down-regulated proteins.

Based on three independent batches of experiments for each group, a total of 132, 122 and 116 proteins were constantly identified at early, middle and late lipid accumulation stages, respectively. At the early lipid accumulation stage, 71 out of the 132 proteins were found to meet the strict criteria for reliable statistical analysis and have considerable fold change (relative expression ≥1.2 or ≤0.8). Majority of these proteins had functions involving in metabolism and ribosomal biosynthesis. At the middle stage, 62 among the 122 proteins passed through the screening; meanwhile, 55 of 116 proteins at the late stage of lipid accumulation went through the filtration. Higher percentages of protein expression in these two stages were found in metabolism, biosynthesis and stress response (Figure 5; Table S1). Fold change of proteins of different functional distribution at 12 h, 24 h and 96 h in nitrogen limited medium was also intuitively figured out in Figure 6. 

Among all the listed proteins, 15 of them were found up-regulated comparing oleaginous yeast strains with *S. cerevisiae*. Five of these 15 proteins were identified in each time point and these included elongation factor 1-alpha, ATP synthase subunit beta, cytochrome c iso-1, 78 kDa glucose-regulated protein and ubiquitin. ADP, ATP carrier protein 2 was only found in 24 h and 96 h samples. Four were identified only in 96 h sample, which were 6-phosphogluconate dehydrogenase decarboxylating 1, NADP-specific glutamate dehydrogenase 2, heat shock protein 70 and thioredoxin-2. The other five proteins were found during 12 h or 24 h samples; including histone H4, heat shock protein HSP90-alpha, phosphomannomutase, luminal-binding protein 4 and cytochrome c. Proteins of particularly importance were illustrated as follows. NADP-specific glutamate dehydrogenase 2 (NADP-GDH3p) is involved in glutamate biosynthesis pathway, catalyzing the reversible reaction between 2-oxoglutarate and glutamate. It was suggested that under low level of nitrogen, this enzyme was found to increase and served as cellular nitrogen supply [30-32]. This would help explain the up-regulation of GDH3p in oleaginous species, especially the unique existence in late lipid accumulation stage, as the nitrogen source was extraordinarily low at 96h. 6-phosphogluconate dehydrogenase, decarboxylating 1 (GND1p) catalyzes the oxidative decarboxylation of 6-phosphogluconate to ribulose 5-phosphate, together with the reduction of NADP to NADPH. The generation of NADPH is critical for varies of reductive biosynthetic reactions, especially lipid production, as well as protecting the cells against oxidative stress [33]. The up-regulation of GND1p in this study was thus important as it indicated sufficient supplement of NADPH was given in oleaginous yeast in lipid accumulation process. ADP, ATP carrier protein 2 (AAC2p) catalyzes the exchange of ADP and ATP across mitochondrial membrane, which imports ADP into mitochondria and pumps the ATP out into cytosol [34]. ATP synthase subunit beta, mitochondrial (ATP2p) converts the energy of proton gradient and produces ATP from ADP across the membrane initiated by electron transport complexes of the respiratory chain [35] and its activity and coupling exhibits strict lipid requirement [36]. The up-regulation of AAC2p and ATP2p in oleaginous yeast strains would not only provide steady supplement of energy, but also was the response for the excess lipid in oleaginous yeast strains. Moreover, higher degree of up-regulation of GND1p, AAC2p and ATP2p were detected in *R. toruloides* comparing with *C. albidus*, which helped explain the higher lipid accumulation ability of *R. toruloides*.

The majority of identified proteins with remarkable change were down-regulated. Interestingly, almost all the enzymes of glycolysis (FBA1p, PYK1p, TDH3p, PGI1p, PGK1p, GLK1p, TPI1p, ENO2p and GPM1p) were found down-regulated during comparison between oleaginous strains and *S. cerevisiae*, as well as two gluconeogenesis proteins (PYC2p and PCK1p). Detailed explanation was difficult, as in the ordinary course of events, glycolysis and gluconeogenesis should be quite active so as to apply sufficient energy source for fatty acid generation. However, this down-regulation would be reasonable when the growth environment difference between oleaginous strains and non-oleaginous one was taken into consideration. As for lipid accumulation, *R. toruloides* and *C. albidus* were transferred from proliferation culture medium into nitrogen-limited medium. The substrate and energy for lipid formation were accumulated gradually in the early phase of growth. Along with lipid accumulation, the metabolic flux was experiencing a shift to lipid biosynthesis. IDH2p, MDH1p, ICL1p and CIT1p were engaged in tricarboxylic acid cycle and also found to be downregulated seriously, indicating the down shift of TCA cycle when cells experiencing lipid accumulation. When oleaginous yeast cells were transferred into nitrogen-limited medium and experienced gradually depletion of nitrogen source, the activity of adenosine monophosphate (AMP) desaminase was sharply increased, leading to the cleavage of AMP [37]. The activity of IDH2p depended on the existence of AMP and was thus degraded critically, leading to a downturn of TCA cycle. This would help explain the down-regulation of enzymes in TCA cycle. Pyruvate decarboxylase isozyme 1 (PDC1p) and alcohol dehydrogenase 1 (ADH1p) catalyzes the pathway from pyruvate to ethanol. They were both down-regulated significantly and it would be easily found that the inhibition of these enzymes contributed to the shift down of the pathways competing with TCA cycle. In other words, the down-regulation of these enzymes in oleaginous yeasts would decrease the energy ‘lose’, lead more metabolites from glucose into TCA cycle, promote pyruvate metabolism and produce more acetyl-CoA, which is the precursor for fatty acid. Meanwhile, it was noticed that most of the proteins with metabolism functions were further down-regulated in *R. toruloides* than *C. albidus*, when comparing with *S. cerevisiae*. This indicated a higher degree of shift from metabolic flux to lipid biosynthesis along lipid accumulation in oleaginous yeast strains.

Many proteins found were involved in stress response, such as heat shock proteins which were found up-regulated, in the early and medium lipid accumulation stage. This would be explained as the cells were grown in a high glucose and low nitrogen medium, which was considered restrictive environment, thus triggering an up-regulation of stress response related proteins. Oleaginous yeast strains produce lipids as a protective mechanism under the stressed circumstances. Meanwhile, at 96 h, it was shown that majority of stress response proteins were down-regulated comparing with those at 12 h or 24 h. The degree of down-regulation was higher in *R. toruloides* than *C. albidus*. This would be clarified as the yeast cells containing high level of unsaturated fatty acid at late stage and decreased stress sensitivity according to M. Chatterjee and S. Khalawan [38]. At late lipid accumulation stage, higher lipid contents were experienced and less stress could be encountered, which explained the down-regulation of stress response proteins in oleaginous yeast.

Numerous ribosomal proteins were also identified, whereas reduced levels of them were reflected over time; most of them were down-regulated during 12 h and 24 h and even undetectable at 96 h, consistent with reduced protein yield along lipid accumulation. The initial period of lipid production should experience the highest protein level, so as to prepare of accumulation of lipid. At late stage, many proteins were degraded and protein synthesis was not as vigorous as starting stage in order to preserve energy for lipid production.

Although the function of proteins with particular importance in lipid accumulation process were discussed above, it is still difficult to explain the functions of some proteins in detail. Also, few proteins were found directly in relate to lipid metabolism. This was understandable given that many of these proteins are hydrophobic and in low abundance, which have poor solubility and separation behaviour. Moreover, the comparison and discussion of proteomic data of these three yeast strains were based on database searched against ‘microorganism’, rather than specific model microbes, remarkable challenges existed for accurate data analysis.

### Influence of Different Culture Conditions

Culture conditions were different for *S. cerevisiae* and the two oleaginous yeast strains. The former was cultured only in rich medium for both growth and lipid production, whereas the latter was transferred to nitrogen limited medium to further trigger lipid accumulation. It was a challenge as a slightly change in the cultural environment would affect the metabolism of yeast cells. However, we tried to culture *S. cerevisiae* using the same nitrogen limited medium of oleaginous yeast strains. They cannot produce lipids normally and experienced poor growth instead, whereas the two oleaginous yeast strains grew vigorously along lipid accumulation. 

When the yeast cells encountered with nitrogen exhaustion, the intracellular AMP (adenosine monophosphate) concentration will be decreased since the AMP-desaminase will cleave AMP into IMP (inosine monophosphate) and NH4+ ions, which could constitute a complementary nitrogen source to support the cell [39]. Meanwhile, the activity of NAD^+^ isocitrate dehydrogenase, which is activated by AMP, will decrease if the intra-cellular AMP concentration drops [40]. As a result, the iso-citric acid, which is found to in equilibrium with citrate, will accumulate inside the mitochondrion. In exchange with malate, citrate will enter the cytoplasm as long as the intra-mitochondrial citric acid concentration reaches a critical value [41]. Citric acid will finally be cleaved to acetyl-CoA and oxaloacetate by ATP-citrate lyase (ATP-CL), generate cellular fatty acids and provide building blocks for lipid accumulation [37,42,43]. However, the ATP-CL gene only exists in oleaginous microorganisms and being absent in the non-oleaginous microbial cells. If ATP-CL enzyme does not exist inside of yeast cells (in our case, the S. *cerevisiae*) which are cultivated in nitrogen limited mediums, the citric acid will either be excreted into culture medium or will provoke the inhibition of the 6-phosphoro-fructokinase, resulting intracellular accumulation of polysaccharides [44]. Therefore, different culture mediums were used to culture the three strains in order to prompt them to produce the highest contents of lipid respectively. Comparison among the strains of low, medium and high lipid accumulation ability at different stages was carried out afterwards. 

## Conclusions

This study successfully set up a platform of time-course proteomic profiling in yeast and to our knowledge, the first one comparing between non-oleaginous with oleaginous yeast strains on proteomics level using iTRAQ-coupled 2-D LC-MS/MS method. It provided an overall reflection from proteomic level, contributing to a better understanding of excessive lipid storage in oleaginous yeast strains and hopefully would contribute to further genetic engineering for higher lipid accumulation in yeast.

## Supporting Information

Table S1 **List of differentially expressed proteins in target yeast strains.**
(DOCX)
