# Supplementary material for: Comparative Proteomics Profile of Lipid-Cumulating Oleaginous Yeast: An iTRAQ-Coupled 2-D LC-MS/MS Analysis
Source: PLoS One. 2013 Dec 26;8(12):e85532. doi: 10.1371/journal.pone.0085532 (PMC3873444; doi:10.1371/journal.pone.0085532)
Supplement: Table S1 — List of differentially expressed proteins in target yeast strains. (DOCX) [file pone.0085532.s001.docx]

**Table S1. List of differentially expressed proteins in target yeast strains.**

| Gene name | Protein name | | Regulation | %AA coverage | MS/MS score | Fold change* | | | | | | | | | |
| --- | --- | --- | --- | --- | --- | --- | --- | --- | --- | --- | --- | --- | --- | --- | --- |
|  |  |  |  |  |  | Early** | | | Middle** | | | | Late** | | |
|  |  |  |  |  |  | 115:114^1)^ | 116:114^2)^ | | 115:114^1)^ | 116:114^2)^ | | 115:114^1)^ | | 116:114^2)^ | |
| **Metabolism** | | | | | | | | | | | | | | | |
| SEC53 | | Phosphomannomutase | Up | 9 | 13 | 0.79±0.04 | | 2.22±0.09 | -- | | -- | -- | | | -- |
| CIT1 | | Citrate synthase, mitochondrial | Down | 26 | 39 | 0.75±0.05 | | 1.78±0.11 | 0.76±0.04 | | 0.57±0.09 | 0.80±0.05 | | | 0.69±0.06 |
| ENO2 | | Enolase 2 | Down | 43 | 181 | 0.18±0.06 | | 0.29±0.02 | 0.26±0.02 | | 0.29±0.04 | 0.87±0.1 | | | 0.42±0.08 |
| FBA1 | | Fructose-bisphosphate aldolase | Down | 11 | 15 | 0.32±0.09 | | 0.35±0.04 | 0.19±0.03 | | 0.25±0.05 | 0.38±0.03 | | | 0.32±0.04 |
| TDH3 | | Glyceraldehyde-3-phosphate dehydrogenase 3 | Down | 52 | 139 | 0.11±0.01 | | 0.21±0.02 | 0.16±0.03 | | 0.19±0.02 | 0.19±0.02 | | | 0.24±0.04 |
| PYK1 | | Pyruvate kinase 1 | Down | 25 | 99 | 0.14±0.03 | | 0.39±0.05 | 0.28±004 | | 0.28±0.03 | 0.31±0.05 | | | 0.19±0.02 |
| PGK1 | | Phosphoglycerate kinase | Down | 26 | 85 | 0.33±0.02 | | 0.30±0.03 | 0.34±0.02 | | 0.37±0.06 | 0.74±0.06 | | | 0.34±0.05 |
| TPI1 | | Triosephosphate isomerase | Down | 32 | 67 | 0.20±0.01 | | 0.44±0.07 | 0.22±0.01 | | 0.29±0.04 | 0.4±0.03 | | | 0.26±0.02 |
| GPM1 | | Phosphoglycerate mutase 1 | Down | 15 | 43 | 0.20±0.03 | | 0.35±0.04 | 0.19±0.06 | | 0.25±0.09 | 0.29±0.02 | | | 0.15±0.01 |
| PGI1 | | Glucose-6-phosphate isomerase | Down | 16 | 53 | 0.28±0.01 | | 0.48±0.05 | 0.36±0.05 | | 0.31±0.07 | 0.46±0.05 | | | 0.30±0.03 |
| GLK1 | | Glucokinase-1 | Down | 7 | 26 | -- | | -- | -- | | -- | 0.40±0.07 | | | 0.17±0.03 |
| PCK1 | | Phosphoenolpyruvate caboxykinase [ATP] | Down | 9 | 35 | -- | | -- | 0.31±0.04 | | 0.32±0.04 | 0.60±0.03 | | | 0.53±0.04 |
| PYC2 | | Pyruvate carboxylase 2 | Down | 14 | 26 | -- | | -- | -- | | -- | 0.49±0.03 | | | 0.5±0.07 |
| PDC1 | | Pyruvate decarboxylase isozyme 1 | Down | 23 | 93 | 0.12±0.01 | | 0.27±0.04 | 0.16±0.01 | | 0.20±0.04 | 0.14±0.02 | | | 0.12±0.02 |
| ADH1 | | Alcohol dehydrogenase 1 | Down | 20 | 23 | 0.19±0.02 | | 0.28±0.05 | 0.42±0.02 | | 0.34±0.03 | 0.36±0.02 | | | 0.2±0.03 |
| OYE2 | | NADPH dehydrogenase 2 | Down | 22 | 42 | 0.34±0.02 | | 0.33±0.03 | 0.42±0.05 | | 0.59±0.06 | -- | | | -- |
| ICL1 | | Isocitrate lyase | Down | 16 | 20 | -- | | -- | 0.31±0.08 | | 0.28±0.03 | 0.34±0.04 | | | 0.21±0.05 |
| MDH1 | | Malate dehydrogenase, mitochondrial | Down | 23 | 23 | -- | | -- | -- | | -- | 0.44±0.09 | | | 0.62±0.08 |
| IDH2 | | Isocitrate dehydrogenase [NAD] subunit 2 | Down | 16 | 20 | -- | | -- | -- | | -- | 0.51±0.07 | | | 0.34±0.02 |
| ADK1 | | Adenylate kinase 1 | Down | 14 | 25 | -- | | -- | 0.36±0.07 | | 0.52±0.09 | -- | | | -- |
| HXK2 | | Hexokinase-2 | Down | 16 | 18 | 0.42±0.04 | | 0.53±0.04 | 0.41±0.04 | | 0.38±0.06 | 0.19±0.03 | | | 0.37±0.09 |
| ALD6 | | Magnesium activated aldehyde dehydrogenae, cytosolic | Down | 15 | 30 | 0.39±0.01 | | 0.72±0.04 | -- | | -- | -- | | | -- |
| IPP1 | | Inorganic pyrophosphatase | Down | 12 | 27 | -- | | -- | 0.51±0.06 | | 0.35±0.03 | 0.28±0.03 | | | 0.24±0.03 |
| HXK1 | | Hexokinase-1 | Down | 6 | 43 | 0.15±0.03 | | 0.44±0.05 | -- | | -- | -- | | | -- |
| HOM6 | | Homoserine dehydrogenase | Down | 10 | 35 | -- | | -- | 0.18±0.02 | | 0.18±0.01 | -- | | | -- |
| ACO1 | | Aconitate hydratase, mitochondrial | Down | 9 | 44 | -- | | -- | 0.33±0.05 | | 0.33±0.05 | -- | | | -- |
| ROCA2 | | 1-pyrroline-5-carboxylate dehydrogenase 2 | Down | 16 | 36 | -- | | -- | -- | | -- | 0.74±0.06 | | | 0.4±0.02 |
| **Biosynthesis** | | | | | | | | | | | | | | | |
| TEF1 | | Elongation factor 1-alpha | Up | 20 | 37 | 1.10±0.04 | | 2.15±0.10 | 1.13±0.05 | | 1.43±0.09 | 1.11±0.11 | | | 1.37±0.08 |
| TMA19 | | Translationally-controlled tumor protein homolog | Down | 20 | 22 | 0.56±0.04 | | 0.43±0.05 | -- | | -- | -- | | | -- |
| ILV5 | | Ketol-acid reductoisomerase, mitochondrial | Down | 18 | 17 | 0.25±0.02 | | 0.42±0.07 | 0.31±0.03 | | 0.27±0.04 | -- | | | -- |
| TEF4 | | Elongation factor1-gamma | Down | 12 | 22 | -- | | -- | -- | | -- | 0.22±0.04 | | | 0.25±0.03 |
| EFB1 | | Elongation factor 1-beta | Down | 11 | 23 | 0.13±0.03 | | 0.26±0.05 | 0.19±0.02 | | 0.21±0.01 | -- | | | -- |
| EFT1 | | Elongation factor 2 | Down | 10 | 25 | 0.28±0.03 | | 0.40±0.02 | 0.85±0.07 | | 0.35±0.06 | 0.18±0.02 | | | 0.06±0.02 |
| YEF3 | | Elongation factor 3A | Down | 19 | 16 | 0.19±0.01 | | 0.56±0.03 | 0.37±0.03 | | 0.45±0.05 | 0.21±0.04 | | | 0.12±0.04 |
| GUS1 | | Glutamyl-tRNA synthetase | Down | 21 | 40 | -- | | -- | -- | | -- | 0.43±0.04 | | | 0.32±0.09 |
| FPP1 | | Farnesyl pyrophosphate synthase | Down | 11 | 31 | -- | | -- | -- | | -- | 0.15±0.02 | | | 0.14±0.04 |
| **Transportation** | | | | | | | | | | | | | | | |
| ATP2 | | ATP synthase subunit beta | Up | 16 | 63 | 2.52±0.07 | | 3.18±0.12 | 2.49±0.08 | | 2.02±0.13 | 1.79±0.14 | | | 2.09±0.11 |
| AAC2 | | ADP, ATP carrier protein 2 | Up | 10 | 17 | -- | | -- | 1.3±0.05 | | 1.74±0.01 | 2.48±0.05 | | | 3.65±0.17 |
| CYCK | | Cytochrome c | Up | 34 | 57 | -- | | -- | 1.17±0.06 | | 0.99±0.07 | -- | | | -- |
| TRX2 | | Thioredoxin-2 | Up | 36 | 38 | -- | | -- | -- | | -- | 2.41±0.09 | | | 1.11±0.05 |
| **Signal transduction** | | | | | | | | | | | | | | | |
| GDH3 | | NADP-specific glutamate dehydrogenase 2 | Up | 24 | 20 | -- | | -- | -- | | -- | 1.74±0.09 | | | 1.34±0.11 |
| GND1 | | 6-phosphogluconate dehydrogenase, decarboxylating 1 | Up | 11 | 43 | -- | | -- | -- | | -- | 1.24±0.08 | | | 1.92±0.09 |
| CYC1 | | Cytochrome c iso-1 | Up | 14 | 26 | 1.47±0.06 | | 1.2±0.07 | 1.36±0.06 | | 1.32±0.08 | 1.63±0.09 | | | 1.15±0.04 |
| PMA2 | | Plasma membrane ATPase 2 | Down | 17 | 26 | 0.29±0.03 | | 0.36±0.03 | 0.64±0.06 | | 0.27±0.03 | 0.60±0.05 | | | 0.5±0.07 |
| POR1 | | Mitochondrial outer membrane protein porin 1 | Down | 13 | 34 | -- | | -- | -- | | -- | 0.17±0.02 | | | 0.18±0.07 |
| NQM1 | | Transaldolase NQM1 | Down | 24 | 27 | -- | | -- | -- | | -- | 0.55±0.07 | | | 3.13±0.21 |
| **Ribosomal proteins** | | | | | | | | | | | | | | | |
| RPS2 | | 40S ribosomal protein S2 | Down | 13 | 39 | 0.21±0.02 | | 0.21±0.01 | 0.21±0.02 | | 0.24±0.03 | 0.21±0.03 | | | 0.29±0.03 |
| RPS7A | | 40S ribosomal protein S7-A | Down | 10 | 28 | 0.23±0.03 | | 0.24±0.07 | 0.22±0.01 | | 0.26±0.04 | 0.53±0.08 | | | 0.43±0.08 |
| RPS5 | | 40S ribosomal protein S5 | Down | 8 | 33 | 0.10±0.04 | | 0.24±0.03 | -- | | -- | -- | | | -- |
| RPS18A | | 40S ribosomal protein S18 | Down | 5 | 30 | 0.16±0.03 | | 0.24±0.02 | -- | | -- | -- | | | -- |
| RPS12 | | 40S ribosomal protein S12 | Down | 16 | 49 | 0.34±0.08 | | 0.55±0.09 | 0.36±0.06 | | 0.29±0.02 | -- | | | -- |
| RPS9B | | 40S ribosomal protein S9-B | Down | 21 | 41 | 0.17±0.02 | | 0.43±0.05 | -- | | -- | -- | | | -- |
| RPS20 | | 40S ribosomal protein S20 | Down | 12 | 19 | 0.18±0.06 | | 0.25±0.04 | -- | | -- | -- | | | -- |
| RPS4A | | 40S ribosomal protein S14 | Down | 14 | 40 | 0.28±0.02 | | 0.52±0.06 | -- | | -- | 0.35±0.07 | | | 0.33±0.08 |
| RPS19A | | 40S ribosomal protein S19-A | Down | 19 | 30 | 0.30±0.06 | | 0.40±0.03 | -- | | -- | -- | | | -- |
| RPS1B | | 40S ribosomal protein S1-B | Down | 22 | 36 | 0.17±0.01 | | 0.48±0.06 | 0.32±0.04 | | 0.29±0.02 | 0.36±0.05 | | | 0.24±0.02 |
| RPS14B | | 40S ribosomal protein S14-B | Down | 13 | 32 | 0.44±0.09 | | 0.57±0.04 | 0.37±0.03 | | 0.46±0.03 | -- | | | -- |
| RPS9A | | 40S ribosomal protein S9-A | Down | 11 | 47 | -- | | -- | -- | | -- | 0.91±0.10 | | | 0.62±0.03 |
| RPS16A | | 40S ribosomal protein S16 | Down | 10 | 34 | 0.08±0.07 | | 0.54±0.05 | -- | | -- | -- | | | -- |
| RPS0 | | 40S ribosomal protein S0 | Down | 6 | 45 | -- | | -- | 0.58±0.06 | | 0.68±0.07 | -- | | | -- |
| RPS3 | | 40S ribosomal protein S3 | Down | 9 | 26 | 0.12±0.03 | | 0.57±0.02 | -- | | -- | -- | | | -- |
| RPS10A | | 40S ribosomal protein S10-A | Down | 12 | 33 | 0.38±0.09 | | 0.66±0.9 | 0.67±0.07 | | 0.49±0.05 | -- | | | -- |
| RPL5 | | 60S ribosomal protein L5 | Down | 15 | 34 | 0.04±0.03 | | 1.63±0.13 | 0.21±0.02 | | 0.60±0.09 | -- | | | -- |
| RPP2A | | 60S acidic ribosomal protein P2-alpha | Down | 20 | 19 | 0.17±0.06 | | 0.31±0.04 | -- | | -- | -- | | | -- |
| RPL15A | | 60S ribosomal protein L15-A | Down | 13 | 21 | 0.30±0.04 | | 0.62±0.09 | -- | | -- | -- | | | -- |
| RPL17A | | 60S ribosomal protein L17-A | Down | 16 | 43 | 0.27±0.03 | | 0.44±0.07 | -- | | -- | -- | | | -- |
| RPL2A | | 60S ribosomal protein L2 | Down | 14 | 39 | 0.65±0.08 | | 0.66±0.08 | -- | | -- | 2.92±0.22 | | | 1.40±0.12 |
| RPL12A | | 60S ribosomal protein L12 | Down | 9 | 22 | 0.25±0.07 | | 0.40±0.05 | 0.26±0.03 | | 0.28±0.07 | -- | | | -- |
| RPL4A | | 60S ribosomal protein L4-A | Down | 10 | 43 | 0.30±0.03 | | 0.56±0.09 | -- | | -- | -- | | | -- |
| RPL3 | | 60S ribosomal protein L3 | Down | 11 | 34 | 0.21±0.04 | | 0.48±0.03 | 0.90±0.08 | | 0.58±0.04 | 0.56±0.04 | | | 0.37±0.08 |
| RPL31B | | 60S ribosomal protein L31-B | Down | 4 | 19 | 0.25±0.06 | | 0.29±0.04 | 0.42±0.05 | | 0.20±0.03 | -- | | | -- |
| RPP0 | | 60S acidic ribosomal protein P0 | Down | 9 | 24 | 0.38±0.09 | | 0.39±0.06 | 0.34±0.04 | | 0.44±0.05 | 0.22±0.07 | | | 0.12±0.04 |
| RPL8B | | 60S ribosomal protein L8-B | Down | 13 | 34 | 0.38±0.10 | | 0.32±0.03 | -- | | -- | -- | | | -- |
| RPL23A | | 60S ribosomal protein L23 | Down | 22 | 22 | -- | | -- | 0.65±0.08 | | 0.56±0.09 | -- | | | -- |
| RPL28 | | 60S ribosomal protein L28 | Down | 20 | 30 | -- | | -- | -- | | -- | 0.82±0.05 | | | 0.37±0.05 |
| RPP2B | | 60S acidic ribosomal protein P2-beta | Down | 12 | 29 | -- | | -- | -- | | -- | 0.44±0.08 | | | 0.31±0.03 |
| **Stress response** | | | | | | | | | | | | | | | |
| HSP90AA1 | | Heat shock protein HSP90-alpha | Up | 12 | 45 | 1.04±0.09 | | 1.55±0.09 | 1.25±0.05 | | 0.93±0.03 | -- | | | -- |
| UBI1 | | Ubiquitin | Up | 19 | 35 | 1.48±0.11 | | 1.35±0.09 | 1.3±0.04 | | 1.35±0.09 | 0.63±0.04 | | | 0.58±0.08 |
| HSA1 | | Heat shock protein 70 | Up | 11 | 23 | -- | | -- | -- | | -- | 1.52±0.07 | | | 0.92±0.05 |
| HSPA5 | | 78kDa glucose-regulated protein | Up | 19 | 46 | 1.51±0.10 | | 1.37±0.07 | 1.71±0.04 | | 1.63±0.02 | 0.84±0.06 | | | 0.65±0.09 |
| AHP1 | | Peroxiredoxin type-2 | Down | 9 | 32 | -- | | -- | 0.06±0.03 | | 0.19±0.02 | 0.25±0.08 | | | 0.1±0.02 |
| TSA1 | | Peroxiredoxin TSA1 | Down | 10 | 24 | 0.33±0.02 | | 0.63±0.07 | 0.45±0.06 | | 0.37±0.06 | 0.45±0.10 | | | 0.37±0.05 |
| HSC82 | | ATP-dependent molecular chaperone HSC82 | Down | 23 | 26 | 0.28±0.02 | | 0.34±0.06 | 0.61±0.08 | | 0.43±0.08 | 0.46±0.09 | | | 0.42±0.08 |
| SSB1 | | Heat shock protein SSB1 | Down | 12 | 32 | 0.26±0.02 | | 0.36±0.03 | 0.5±0.09 | | 0.52±0.09 | 0.54±0.01 | | | 0.26±0.03 |
| SSA2 | | Heat shock protein SSA2 | Down | 14 | 22 | -- | | -- | 0.75±0.04 | | 0.85±0.11 | -- | | | -- |
| SSC1 | | Heat shock protein SSC1 | Down | 19 | 13 | -- | | -- | 0.67±0.06 | | 0.61±0.04 | 0.51±0.09 | | | 0.51±0.05 |
| HSP12 | | 12 kDa heat shock protein | Down | 9 | 19 | -- | | -- | 0.24±0.03 | | 0.25±0.06 | 0.43±0.08 | | | 0.43±0.04 |
| HSC71 | | Heat shock protein cognate 70kDa protein | Down | 6 | 11 | 0.46±0.05 | | 0.93±0.09 | 1.35±0.08 | | 0.68±0.07 | -- | | | -- |
| HSP26 | | Heat shock protein 26 | Down | 11 | 32 | -- | | -- | 0.21±0.06 | | 0.36±0.02 | -- | | | -- |
| SSE1 | | Heat shock protein homolog SSE1 | Down | 16 | 16 | 0.16±0.01 | | 0.39±0.04 | -- | | -- | -- | | | -- |
| GPP1 | | (DL)-glycerol-3-phosphatase 1 | Down | 11 | 22 | 0.48±0.07 | | 0.6±0.09 | 0.48±0.05 | | 0.38±0.04 | -- | | | -- |
| **Structural proteins** | | | | | | | | | | | | | | | |
| HHF1 | | Histone H4 | Up | 10 | 26 | 0.6±0.05 | | 1.28±0.06 | 2.13±0.07 | | 1.78±0.05 | -- | | | -- |
| HTB1 | | Histone H2B | Down | 17 | 27 | 0.42±0.06 | | 0.69±0.08 | 0.89±0.06 | | 1.15±0.07 | -- | | | -- |
| **Others** | | | | | | | | | | | | | | | |
| BIP4 | | Luminal-binding protein 4 | Up | 32 | 34 | -- | | -- | 0.89±0.03 | | 1.15±0.08 | -- | | | -- |
| ASC1 | | Guanine nucleotide-binding protein subunit β protein | Down | 8 | 12 | 0.10±0.04 | | 0.38±0.03 | -- | | -- | -- | | | -- |
| CPR1 | | Peptidyl-prolyl cis-trans isomerase | Down | 9 | 33 | 0.86±0.08 | | 0.28±0.02 | 0.37±0.03 | | 0.61±0.07 | 0.17±0.02 | | | 0.21±0.02 |
| TIF1 | | ATP-dependent RNA helicase elF4A | Down | 8 | 31 | 0.16±0.01 | | 0.35±0.04 | -- | | -- | -- | | | -- |
| HYP2 | | Eukaryotic translation initiation factor 5A-2 | Down | 9 | 21 | 0.20±0.02 | | 0.45±0.05 | 0.36±0.02 | | 0.34±0.02 | -- | | | -- |
| STM1 | | Suppressor protein STM1 | Down | 14 | 30 | 0.27±0.04 | | 0.38±0.06 | -- | | -- | -- | | | -- |
| CDC48 | | Cell division control protein 48 | Down | 13 | 29 | 0.22±0.03 | | 0.39±0.07 | -- | | -- | 0.74±0.07 | | | 0.54±0.05 |
| dnaE | | DNA polymerase III subunit alpha | Down | 10 | 20 | 0.6±0.09 | | 0.86±0.09 | -- | | -- | -- | | | -- |
| CYS3 | | Cystathionine gamma-lyase | Down | 8 | 32 | 0.16±0.04 | | 0.97±0.1 | -- | | -- | -- | | | -- |
| ZEO1 | | Protein ZEO1 | Down | 11 | 39 | 0.12±0.01 | | 0.42±0.07 | 0.36±0.03 | | 0.23±0.05 | -- | | | -- |
| BMH2 | | Protein BMH2 | Down | 15 | 28 | 0.69±0.08 | | 0.71±0.05 | 0.32±0.04 | | 0.24±0.03 | -- | | | -- |
| SOD2 | | Superoxide dismutase [Mn], mitochondrial | Down | 5 | 39 | -- | | -- | 0.62±0.07 | | 0.29±0.04 | -- | | | -- |
| DUG1 | | Cys-Gly metallodipeptidase | Down | 14 | 37 | -- | | -- | -- | | -- | 0.24±0.03 | | | 0.28±0.05 |

*Data represented mean±S.D. based on three independent replicate experiments. “—” indicated the protein was absent.

**Early, Middle and Late indicated samples were from early, middle and late lipid accumulation stages.

1) The ratio of protein expression level in *C. albidus* (115) to *S. cerevisiae* (114).

2) The ratio of protein expression level in *R. toruloides* (116) to *S. cerevisiae* (114).
